# Novel long non-coding RNAs associated with inflammation and macrophage activation in human

**DOI:** 10.1038/s41598-023-30568-1

**Published:** 2023-03-10

**Authors:** Avisankar Chini, Prarthana Guha, Venkat S. Malladi, Zibiao Guo, Subhrangsu S. Mandal

**Affiliations:** 1 Department of Chemistry and Biochemistry, The University of Texas at Arlington, Arlington, TX 76019 USA; 2 North Texas Genome Center, The University of Texas at Arlington, Arlington, TX 76019 USA; 3 Lyda Hill Department of Bioinformatics, Bioinformatics Core Facility, University of Texas Southwestern Medical Center, Dallas, TX 75390 USA

**Keywords:** Biochemistry, Genetics

## Abstract

Inflammation plays a central role in immune response and macrophage activation. Emerging studies demonstrate that along with proteins and genomic factors, noncoding RNA are potentially involved in regulation of immune response and inflammation. Our recent study demonstrated that lncRNA HOTAIR plays key roles in cytokine expression and inflammation in macrophages. The primary goal of this study is to discover novel lncRNAs that are crucial players in inflammation, macrophage activation, and immune response in humans. Towards this, we have stimulated THP1-derived macrophages (THP1-MΦ) with lipopolysaccharides (LPS) and performed the whole transcriptome RNA-seq analysis. Based on this analysis, we discovered that along with well-known marker for inflammation (such as cytokines), a series of long noncoding RNAs (lncRNAs) expression were highly induced upon LPS-stimulation of macrophages, suggesting their potential roles in inflammation and macrophage activation. We termed these family of lncRNAs as Long-noncoding Inflammation Associated RNA (LinfRNA). Dose and time dependent analysis demonstrated that many human LinfRNA (hLinfRNAs) expressions follow similar patterns as cytokine expressions. Inhibition of NF-κB suppressed the expression of most hLinfRNAs suggesting their potential regulation via NF-κB activation during inflammation and macrophage activation. Antisense-mediated knockdown of hLinfRNA1 suppressed the LPS-induced expression of cytokines and pro-inflammatory genes such as IL6, IL1β, and TNFα expression, suggesting potential functionality of the hLinfRNAs in cytokine regulation and inflammation. Overall, we discovered a series of novel hLinfRNAs that are potential regulators of inflammation and macrophage activation and may be linked to inflammatory and metabolic diseases.

## Introduction

Inflammation is a vital biological process associated with the immune response^1–3^. The immune system recognizes and removes injurious stimuli (e.g. bacterial and viral infections) and helps healing. Inflammation causes activation of immune cells such as monocytes, macrophages, T cells, and others and this induces production of inflammatory mediators such as cytokines and chemokines, to fight against infection. However, uncontrolled and chronic inflammation and dysregulation in macrophage activation contribute to many human severe diseases including sepsis, autoimmune disorders, atherosclerosis, metabolic diseases, neurological disorders and cancer^4–9^. Despite the extensive research and availability of a wide variety of drugs, many chronic and inflammatory diseases cannot be treated effectively. Therefore, finding novel therapeutic targets and discovering critical regulators of inflammation for developing effective therapeutic strategies becomes urgent.

Macrophage activation plays a central role in inflammation and immune response^10,11^. Macrophages are originally derived from bone marrow derived monocytes and then infiltrate through blood vessels to reach different tissues for protecting against different kinds of tissue damage and infections. Once reached to different tissue, macrophages differentiate into tissue specific resident macrophages such as Kupffer cells, microglia etc. Resting macrophages (M0 type macrophages) produce very low levels of cytokines and inflammatory mediators^10–12^. However, upon inflammation, tissue resident or recruited macrophages (from circulatory macrophages) gets activated by different types of inflammatory mediators and differentiate into M1 (pro-inflammatory or killer macrophage) and M2 (ant-inflammatory or healing macrophages) type of macrophages^10–13^. M1 macrophages secrete various cytokines, chemokine and pro-inflammatory signals and induces inflammatory response and that helps removing pathogenic infection and phagocytosis. M2-macrophages secrete high levels of anti-inflammatory cytokine (such as IL4, IL10, IL13, etc.) and helps wound healing and tissue regeneration. Under chronic inflammation macrophages cause tissue damage and metabolic reprogramming contributing toward chronic metabolic diseases such as obesity, diabetes, cardiovascular diseases, neurological disorder, and multiorgan failure.

Inflammation and immune signaling follow complex pathways involving various genomic and protein-based factors (e.g. cytokines, interferons, etc.)^14–18^. However, emerging evidence suggests that along with proteins, noncoding RNAs (ncRNAs) are key players in the immune response and inflammation^19–25^. For example, miR-155 is induced upon inflammation via targeting IκB in NF-κB activation^26,27^. Similar to microRNAs, Lethe, a long noncoding RNA (lncRNA), is induced upon TNFα stimulation and it inhibits NF-κB by interacting with RelA (P65) subunit of active NF-κB^28^. LincRNA-Cox2 inhibits interferon-stimulated genes and chemokines in resting macrophages (M0)^29^. LncRNA P50-associated COX-2 extragenic RNA (PACER) has been reported as a regulator of NF-κB signaling and PTGS2 (COX-2) expression^30^. LncRNA THRIL has been reported a negative feedback regulator of TNFα. The lncRNA NEAT1 (Nuclear enriched abundant transcript 1), is involved in controlling the heterochromatin structure formation and virus medicated pathogenesis and immunity^31^.

Recently, we discovered that lncRNA HOTAIR plays a critical role in inflammation^32,33^. Notably, HOTAIR, is one of the most well-studied LncRNAs, which regulates gene silencing via coordination with PRC2 and LSD1 complexes^34–37^. Our studies showed that, beyond its classical roles in gene silencing, HOTAIR regulates the expression of proinflammatory cytokines, glucose transporter, glucose uptake, and glucose metabolism in macrophages during inflammation and this is mediated via regulation of NF-κB activation. Taken together, our findings demonstrated the critical roles of HOTAIR in inflammation, macrophage activation, and immune response^32,33^. Thus, LncRNAs appear to be integral component of inflammation and immune response. Here we aimed to discover novel LncRNAs that are critical regulators of inflammation and immune signaling in humans in an unbiased manner and explored their potential functions and regulations. For discovering human specific LncRNAs associated with inflammation, we used macrophages derived from THP1 cells (monocytes, human acute leukemia cells). Notably, THP1-derived macrophages are widely used as a model cell line^38–40^ for human macrophages for studying inflammation and immune response.

## Materials and methods

### Cell culture, macrophage differentiation, and treatment with Lipopolysaccharide (LPS)

Human acute leukemia cells (THP1, monocyte, ATCC) were cultured and maintained in T75 cell cultured flasks using RPMI-1640 media, supplemented with 10% heat-inactivated FBS (fetal bovine serum), 2 mM L-glutamine, 100 units/mL penicillin and 0.1 mg/mL streptomycin) in a humidified incubator with 5% CO_2_ and 95% air at 37 °C^41–43^.

### Differentiation of THP1 cells (monocytes) into macrophages

THP1 cells were grown in 60 mm cell culture plate using complete RPMI-1640, treated with 25 nM of Phorbol 12-myristate 13-acetate (PMA, stock solution in DMSO) for 48 h^42,43^. Media was replaced with complete RPMI-1640 medium without PMA and incubated for additional 24 h for the recovery and this resulted in differentiated THP1-derived macrophage (THP1-MΦ). Notably, THP1 monocytes grow in suspension, however, after differentiation into macrophages, they become adherent. THP1-MΦ were further characterized by immunostaining for expression of surface antigens (such as CD68).

### LPS-treatment

3 × 10^6^ cells THP1 cells were seeded in 60 mm cell culture dishes, differentiated into THP1-MΦ using PMA (as above) and then treated with LPS (1.0 μg/mL, Invivogen) for 4 h (or varying dose or time periods) and subjected to RNA and protein extraction, and immunostaining, as needed^32,33^.

### RNA-sequencing (RNAseq) analysis

#### RNA extraction

Total RNA was extracted from cells cultured in 60 mm dish using RNeasy RNA extraction kit (Qiagen) following the manufacturer’s protocol. The final RNA was eluted and quantified using nanodrop spectrophotometer. Prior to making the libraries, RNA concentration was again measured on a Qubit 4.0 Fluorometer (Thermo Fisher Scientific, USA) using Qubit RNA BR Assay Kit (Cat# Q10210, Thermo Fisher Scientific, USA). RNA quality was assessed on an Agilent Biotechnologies 4200 TapeStation (Agilent Technologies, USA) using RNA ScreenTape (Cat# 5067–5576), and average RIN was 8.7.

#### *Library preparation and transcriptome sequencing*^44–46^

The input for library construction was 500 ng of total RNA which was delivered in a 10 µL volume. Then total RNA samples (500 ng) were hybridized with Ribo-Zero Gold to substantially deplete cytoplasmic and mitochondrial rRNA from the samples. Stranded RNA sequencing libraries were prepared as described using the Illumina TruSeq Stranded Total RNA Library Prep Gold kit (Cat# 20020598, Illumina, USA) with IDT-for Illumina TruSeq RNA UD Indexes (Cat# 20023785, Illumina, USA). The average insert size of libraries constructed with the TruSeq Stranded Total RNA Library Prep Kit was 200 bp. Purified libraries were qualified on an Agilent Technologies 4200 TapeStation using a D1000 ScreenTape assay (cat# 5067–5582). The molarity of adapter-modified molecules was defined by quantitative PCR using the Kapa Biosystems Kapa Library Quant Kit (Cat#KK4824, KAPA). Individual libraries were normalized to 1.30 nM in preparation for Illumina sequence analysis. Sequencing libraries (1.3 nM) were chemically denatured and applied to an Illumina NovaSeq flow cell using the NovaSeq XP chemistry workflow (Cat# 20021664, Illumina, USA). Following transfer of the flow cell to an Illumina NovaSeq instrument, a 2 × 151 cycle paired end sequence run was performed using a NovaSeq S4 reagent Kit (Cat# 200122866, Illumina, USA).

#### *Analysis of RNA-seq data*^47,48^

Reads with phred quality scores less than 20 and less than 35 bp after trimming were removed from further analysis using trimgalore (v0.4.1). Quality-filtered reads were then aligned to the reference genome using the HISAT (v 2.0.1) PMID: 27560171) aligner using default settings and marked duplicates using Sambamba (v0.6.6) (PMID: 25697820). Aligned reads were quantified using ‘featurecount’ (v1.4.6) (PMID: 30783653) per gene ID against GENCODE (PMID: 30357393). Differential gene expression analysis was done using the R package edgeR (v3.10.5) (PMID:19910308)^49,50^. Cutoff values of absolute fold change greater than 2.0 and FDR ≤ 0.05 were then used to select for differentially expressed genes between sample group comparisons.

#### *RNA extraction, cDNA synthesis, and real-time PCR*^32,33^

Total RNA was extracted from the control and treated THP1-MΦ cells using TRIzol™ Reagent (Invitrogen) according to the manufacturer’s instructions. Briefly, supernatant from the cell culture plates were discarded after treatment, then 500 μL of Trizol reagent were directly added into the plate containing cells, incubated for 10 min. Cell lysate was harvested into 1.5 mL Eppendorf tube and further incubated on ice for 30 min with occasional mixing. Chloroform (100 μL, one fifth volume of Trizol reagent) was added to the cell lysate, mixed, incubated (15 min, on ice) and then centrifuged at 12,000 rpm for 15 min (at 4 °C). The top aqueous layer was collected carefully, mixed with equal volume of isopropanol (10 min, rt) and centrifuged at 12,000 rpm (10 min, at 4 °C). The precipitated RNA pellet was washed with 70% ethanol (ice cold), air dried, and finally the RNA was dissolved in 50 μL of RNase-free water (DEPC treated, Sigma) and quantified using a Nanodrop spectrophotometer.

#### cDNA synthesis

cDNA synthesis (reverse transcription) was performed in two steps, RT1 and RT2. In RT1, 1 μg of total RNA was mixed with 0.6 μL of oligo (dT)_15_ primer (Promega, 500 μg/mL stock) and RNase free water to a final volume of 12 μL, and incubated for 15 min at 70 °C. In RT2, 5 μL of 5 X M-MLV RT Buffer (Promega), 2 μL of DTT (10 mM DTT stock in nuclease free water), 0.25 μL of dNTP Mix (40 mM stock, Promega), 0.25 μL of RNase Inhibitor (40 U/μL, Promega), 0.5 μL of M-MLV Reverse Transcriptase (200 U/μL, Promega) was mixed with nuclease free water to a final volume of 13 μL and then combined with RT1 (after the initial incubation). For the cDNA synthesis, the RT1 and RT2 mix (total volume 25 μL) was incubated for 90 min at 37 °C, followed by 5 min incubation at 95 °C, and then at 4 °C (final hold), in a thermocycler. Finally, the cDNA was diluted in nuclease free water to a final volume of 100 μL.

#### qPCR analysis

The qPCR was performed in CFX96 real-time detection system (Bio-Rad), using iTaq Universal SYBR Green Supermix (Bio-Rad) with gene specific PCR primers as listed in Table 1. In brief, 2 µL of cDNA (template) was mixed with 1 µL of gene specific primer pair (0.5 µM final concentration for both forward and reverse), 3 µL of iTaq Universal SYBR Green Supermix, and nuclease free water to a final volume of 10 µL. The polymerase chain reaction was programmed for an initial denaturation at 95 °C for 2 min and in-loop denaturation at 95 °C for 5 s, both annealing and polymerization combined for 30 s at 58 °C for 39 cycles. The threshold fluorescence (RFU) was determined by the CFX96 real-time detection system (Bio-Rad) software. The threshold cycles (C_t_) of each expression data were normalized to the corresponding β-Actin expression and expressed as 2^(−ΔCt)^. Each qPCR analysis was performed in three parallel replicates and the experiment was repeated thrice.

**Table 1:** qPCR Primer sequences Forward (5′-3′) Reverse (5′-3′).

Gene	Forward (5’ → 3’)	Reverse (5’ → 3’)
Gene specific qPCR primers		
β-Actin	CTCTTCCAGCCTTCCTTCCT	AGCACTGTGTTGGCGTACAG
IL6	GAAAGCAGCAAAGAGGCACT	TTTCACCAGGCAAGTCTCCT
IL1β	AAGGCGGCCAGGATATAACT	CCCTAGGGATTGAGTCCACA
ACOD1	GGTTTTCTCCAGTGCCCATA	CAACTTGCCAAGCTTCAACA
IDO1	TCAGTGCCTCCAGTTCCTTT	CCTGAGGAGCTACCATCTGC
TNFAIP6	CAACTCTGCCCTTAGCCATC	AAGCTCACCTACGCAGAAGC
CXCL11	TGGGATTTAGGCATCGTTGT	CCTGGGGTAAAAGCAGTGAA
DLL4	ACAGTAGGTGCCCGTGAATC	GCGAGAAGAAAGTGGACAGG
ADORA2A-AS1	TCATGGTGAAGGGTGATGAA	GCTCAGAAAGCTTGGACACC
AC007362.3	GGCTTTGGATGGTTGAAGAG	TCCCCTAAGCTCCTTCCTGT
AP001610.5	GCACGTTCTCTCCCCAAATA	CTTCAGGTGGAACACGAGGT
RP11-519G16.3	GGGAAATTCCATGGTTTCCT	GGGTCCTCAAATCAGCTGTC
RP1-68D18.4	GCGCTGTGGTCCAATAGACT	CCAGAGGACAAAAGGCAAAA
Antisense oligonucleotides (ASO) and Scramble oligonucleotide sequences (5′-3′)		
hLinfRNA1– ASO1	GGCAGATCTCTTCACTCCAGA	
hLinfRNA1 –ASO2	TTCGTAGACAAGCATGTGGTG	
hLinfRNA1 –ASO3	TCTTCCTCGGTAGTCCTGTGA	
Scramble oligo	TCCATGGCCAACACTTGTCA	

#### *Protein extraction and western blot analyses*^32,33^

The cells were washed twice in ice-cold PBS, mixed with RIPA cell lysis buffer (Thermo Fisher), complete protease inhibitor cocktail (1 X), and phosphatase inhibitor (1X) cocktail (Roche) and incubated 30 min on ice. The resulting cell lysates were centrifuged at 13,000 rpm at 4 °C for 10 min. The supernatant was collected, and protein was quantified using BCA protein assay kit (Pierce). For the Western blot, 30 μg protein was loaded onto 10% SDS-PAGE gels, transferred to nitrocellulose membrane, and blocked with 5% nonfat dry milk (in 1 X TBST). Membranes were washed (thrice, 1 X TBST, 5 min each), incubated with primary antibodies against Phospho-IκBα (1:1000 dilution, 2859S, Cell Signaling), Phospho-p65 (NF-κB subunit, 1:1000 dilution, 3033S, Cell Signaling), IκBα (1:1000 dilution, 4814 T, Cell Signaling), p65 (1:1000 dilution, 10745-1-AP, Proteintech), IL6 (1:1000 dilution, GTX110527, GeneTex), ACOD1 (1: 1000 dilution, 775010S, Cell Signaling), and IDO1 (1:1000 dilution, 13268-1-AP, Proteintech), IL1 β (16806-1-AP, Proteintech) and β-actin (1:1000 dilution, A2066, Sigma) overnight at 4 °C. Membranes were washed (thrice) and incubated with AP-conjugated Goat anti-mouse (# ab97020, Abcam) or goat anti-rabbit secondary (# ab6722, Abcam) antibodies for 2 h. Membranes were washed (thrice) and developed with BCIP-NBT (Alkaline phosphatase substrate) solution. For ECL Western blot, we used Horseradish peroxidase conjugated goat Anti-Mouse (# ab6789, Abcam) or goat Anti-Rabbit (# ab6721, Abcam) secondary antibodies and developed with developed using Pierce ECL western blotting substrate in LI-COR C-Digit blot scanner. Bands were quantified with ImageJ software and plotted.

#### *NF-κB inhibition assay*^32,33^

THP1 cells (3 × 10^6^) were seeded in 60 mm cell culture dishes and differentiated into macrophages (THP1-MΦ, using PMA as above). After differentiation, cells were initially treated with IKKβ inhibitor (SC-514, 25 μM, Sigma) for 1 h (to inhibit NF-kB signaling) followed by treatment with LPS (1 μg/mL) for 4 h. RNA and proteins were isolated and analyzed by RT-qPCR and Western blotting. For the phospho-protein analysis, cells were harvested at 1 h post LPS-treatment and proteins were isolated and probed for Western blotting.

#### Immunofluorescence microscopy analysis

THP1 cells were seeded on cover slips and differentiated into macrophage (THP1-MΦ), fixed in 4% paraformaldehyde (PFA, 10 min at rt), washed (1 X PBST, thrice 5 min each), permeabilized with 0.1% triton X100 (15 min, 1 X PBST, rt), and blocked with 3% BSA (in 1XPBST for 1 h). The cells were then incubated with mouse anti-human CD68 primary antibody (# 14-0688-82, Invitrogen, 4 h, rt). Cells were washed 3 times with PBST followed by incubation with FITC-conjugated goat Anti-Mouse (# 6785, Abcam) secondary antibodies for 1 h at RT. DAPI was added to the cells with a final concentration of 1 μg/mL and incubated for 15 min. Finally, the cells were washed 3 times with PBST and fixed with mounting media on a slide and analyzed under a fluorescence microscope (Nikon ECLIPSE TE2000-U).

### Antisense-mediated knockdown of hLinfRNA1

THP1 cells (1 million/well) was seeded in a 6-well plate and differentiated into THP1-MΦ cells using PMA (as described above). The media was exchanged with fresh complete RPMI prior and then subjected to transfection with LinfRNA1-specific and scramble antisense oligonucleotide (ASO) using similar protocols described earlier^51–53^. Briefly, transfection cocktail was prepared by mixing 1.5 μg of ASO (diluted with 100 μL of 1X transfection buffer) with 3 μL of transfection reagent (GeneMute, SignaGen) at room temperature for 15 min. The cocktail was added to the cells dropwise and gently mixed throughout the well by swirling. After 5 h of incubation, the cultured media was replaced by 2 mL of complete media and cells were further incubated for 43 h, then treated with LPS (1 μg/mL) and incubated for additional 4 h. Control and transfected cells were harvested and subjected to RNA purification using Trizol reagent. RNA was reverse transcribed and subjected qPCR analysis using primers specific to hLinfRNA1 and others. Human beta-actin was used as loading control.

### Statistical analysis

Each experiment was performed three times with at least three replicates (n = 3) independently. Data are presented as means ± SEM. All Statistical significance was calculated by unpaired Student’s t test (GraphPad Prism 6), and *P *≤ 0.05 was considered statistically significant.

## Results

### Differentiation of THP1 cells into corresponding macrophages and analyzing their inflammatory response

**Figure 1:**
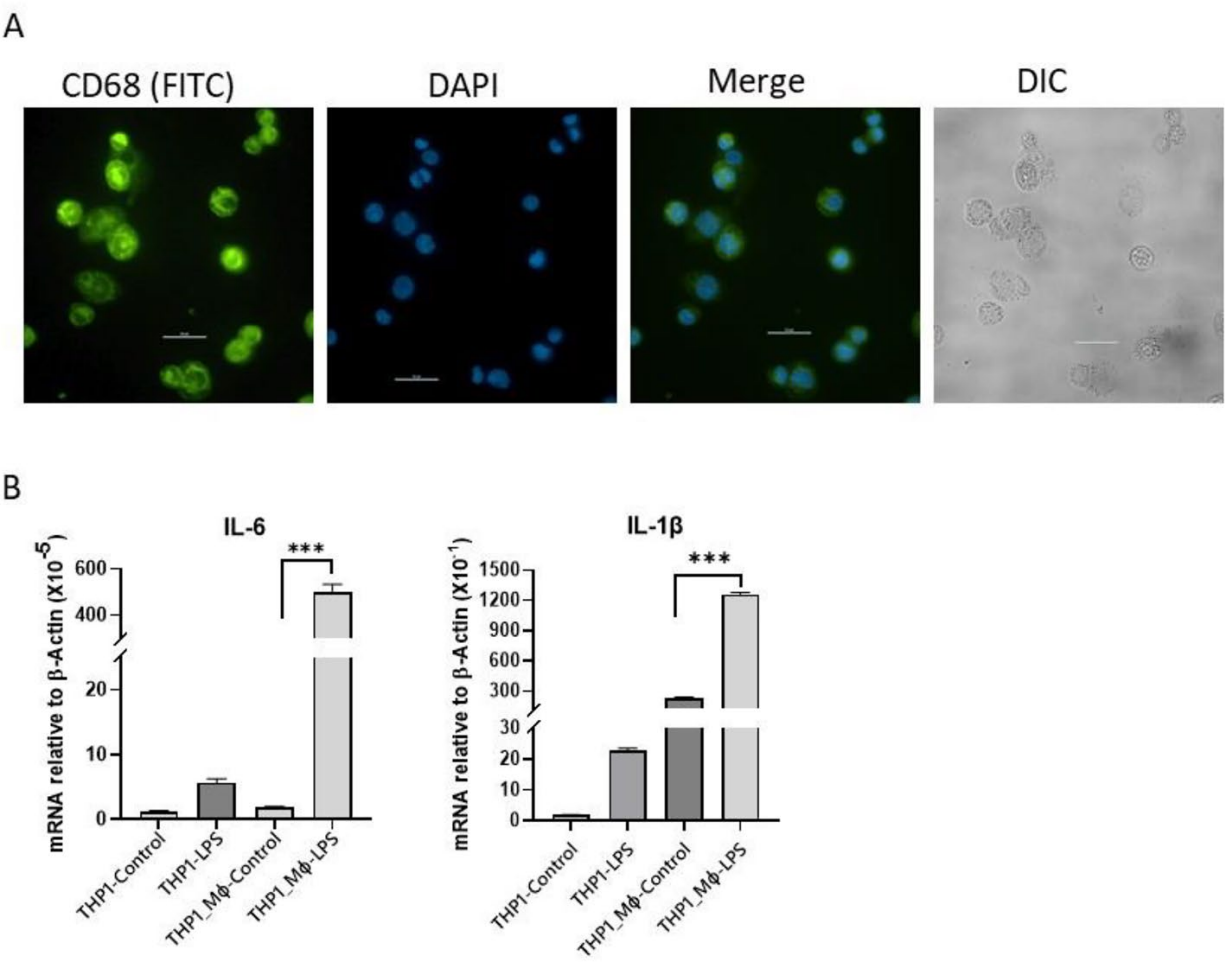
PMA induced differentiation of THP1 monocytes into macrophages (THP1-Mɸ) and their response to LPS-stimulation. (**A**) THP1 cells (monocytes) were differentiated using PMA (25 nM, 72 h) on a coverslip (35 mm cell culture plate). Cells were immuno-stained with CD68 antibody (mouse) followed by FITC-conjugated secondary antibodies, counterstained with DNA binding dye DAPI, mounted, and analyzed under a fluorescence microscope. Images taken at 40X resolution (bar = 50 μm). (**B**) LPS-stimulation of THP1 (monocytes) and THP1-Mɸ. THP1 and THP1-Mɸ cells were treated with LPS (1 μg/mL, 4 h) independently, total RNA was isolated, reverse transcribed to cDNA, and analyzed by RT-qPCR for expression of IL-6 and IL-1β. Each experiment was repeated at least thrice with three parallel replicates. β-Actin was used as loading control. Data represent mean ± SEM (n = 3); *p < 0.05, **p < 0.001, ***p < 0.0001.

To discover LncRNAs that are associated with inflammation and immune response in an unbiased manner, we performed RNA-seq analyses in human THP1 derived macrophage (THP1-MΦ) cells that were stimulated with LPS. Prior to LPS stimulation, THP1 cells were differentiated in macrophages as described previously^42,43^. Briefly, the THP1 cells were treated with 25 nM PMA for 48 h followed 24 h recovery. The differentiation of THP1 cells into macrophages (THP1-MΦ) were confirmed by analyzing the expression of macrophage specific surface marker antigen (CD68) using immunofluorescence microscopy (Fig. 1A). Briefly, THP1-MΦ were immune-stained with anit-CD68 antibody followed by immunostaining with FITC- tagged secondary antibody. The immuno-fluorescence microscopic analysis showed that CD68 is expressed at the membrane surfaces suggesting successful differentiation of the THP1 monocytic cells into macrophages. We also examined the inflammatory response of control (undifferentiated THP1) with differentiated THP1-MΦ. Briefly, undifferentiated THP1 and THP1-MΦ were independently treated with endotoxin lipopolysaccharide (LPS, 1 µg/mL, 4 h) and then expression of well-known pro-inflammatory markers such as interleukin 6 (IL6) and IL1β were analyzed using RT-qPCR. Our analysis showed that, LPS treatment induced the expression of both IL6 (250-fold) and IL1β (sixfold) mRNA in THP1-MΦ macrophages (Fig. 1B). However, LPS has no significant impacts on IL6 and IL1β expression in undifferentiated THP1 (monocytes) cells. These observations further demonstrated that PMA treatment indeed resulted in differentiation of THP1 cells (monocytes) into corresponding macrophages (THP1-MΦ) and macrophages are responsive to inflammatory stimuli such as LPS-treatment. Notably, the THP1 cells (monocytes) that grew in suspension culture became adherent upon differentiation to macrophages with PMA. Any nonadherent cells were removed during media replacement and the differentiated THP1-MΦ were used for the further experiments.

### RNA-seq analysis to identify LncRNAs associated with inflammation

**Figure 2:**
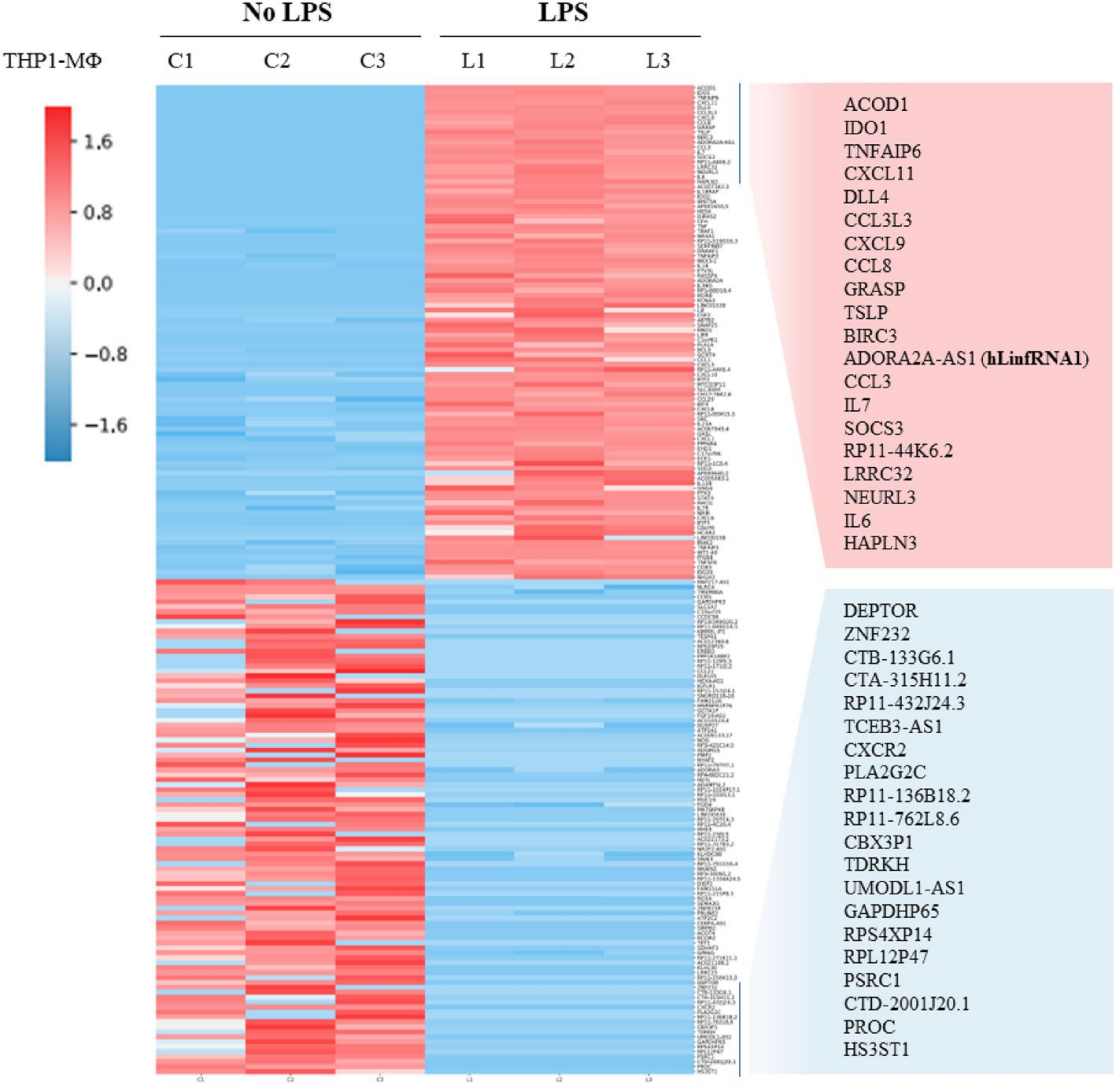
RNAseq analysis of LPS-treated macrophages. THP1** c**ells were differentiated into PMA into macrophages (THP1-MΦ), treated with LPS (1.0 μg/mL) for 4 h. Total RNA was extracted from the control cells (C1–C3) and LPS-treated THP1-MΦ cells (L1–L3), quantified and subjected to ribo-depletion followed by library construction using the Illumina TruSeq Stranded Total RNA Library Prep Gold kit. Libraries were sequenced in an Illumina NovaSeq instrument. Differential gene expression analysis was done using the R package edgeR (v3.10.5) (PMID:19910308). Differentially expressed genes (log2 -old) were plotted as a heatmap. Cutoff values of absolute fold change greater than 2.0 and FDR ≤ 0.05 were used to select for differentially expressed genes between sample group comparisons.

To identify LncRNAs associated with inflammation, we performed RNAseq analysis in THP1-MΦ that were stimulated with LPS. Briefly, THP1-MΦ cells were treated with LPS (1 µg/mL, 4 h). RNA was isolated from control (untreated) and LPS-treated THP1-MΦ, quantified and subjected to RNAseq analysis. Briefly, total RNA from the control and LPS-treated THP1-MΦ isolated and subjected to ribodepletion using RiboZero Gold kit to substantially deplete cytoplasmic and mitochondrial rRNA from the samples. Stranded RNA sequencing libraries were prepared using the Illumina TruSeq Stranded Total RNA Library Prep Gold kit and the average insert size of libraries constructed was 200 bp. Purified libraries were qualified, normalized (to 1.30 nM), and then applied to RNAseq analysis using Illumina NovaSeq instrument. Data analyses were performed at the UT Southwestern’s bioinformatics core facility and differentially expressed genes were compared and plotted. These analysis demonstrated that LPS-stimulation of THP1-MΦ resulted in upregulation of many well-known markers of inflammation, these include proinflammatory cytokines IL6 (interleukin 6), TNFα (tumor necrosis factor alpha), CXCL (chemokines), CL(chemokines), and others^54,55^ (Fig. 2). Additionally, we also found up- and down-regulation of many protein coding genes such as ACOD1 (aconitate decarboxylase 1)^56,57^, IDO1 (Indoleamine 2,3-Dioxygenase 1)^58–60^, and others (Fig. 2) under LPS treatment conditions suggesting their potential association with inflammation and immune response. Notably, ACOD1, also known as IRG1 (immune responsive gene 1) is previously implicated in inflammation and immune response)^56,57^. Similarly, IDO1, a heme-based enzyme, is a well-known player in tryptophan catabolism and inflammation^61–64^. IDO1 is upregulated in inflammatory diseases and is a major drug target for immunotherapy. Thus, LPS-induced upregulation of cytokines and well-known proinflammatory genes suggested the potential functionality of other novel genes that are identified based on our RNAseq analysis.

**Figure 3:**
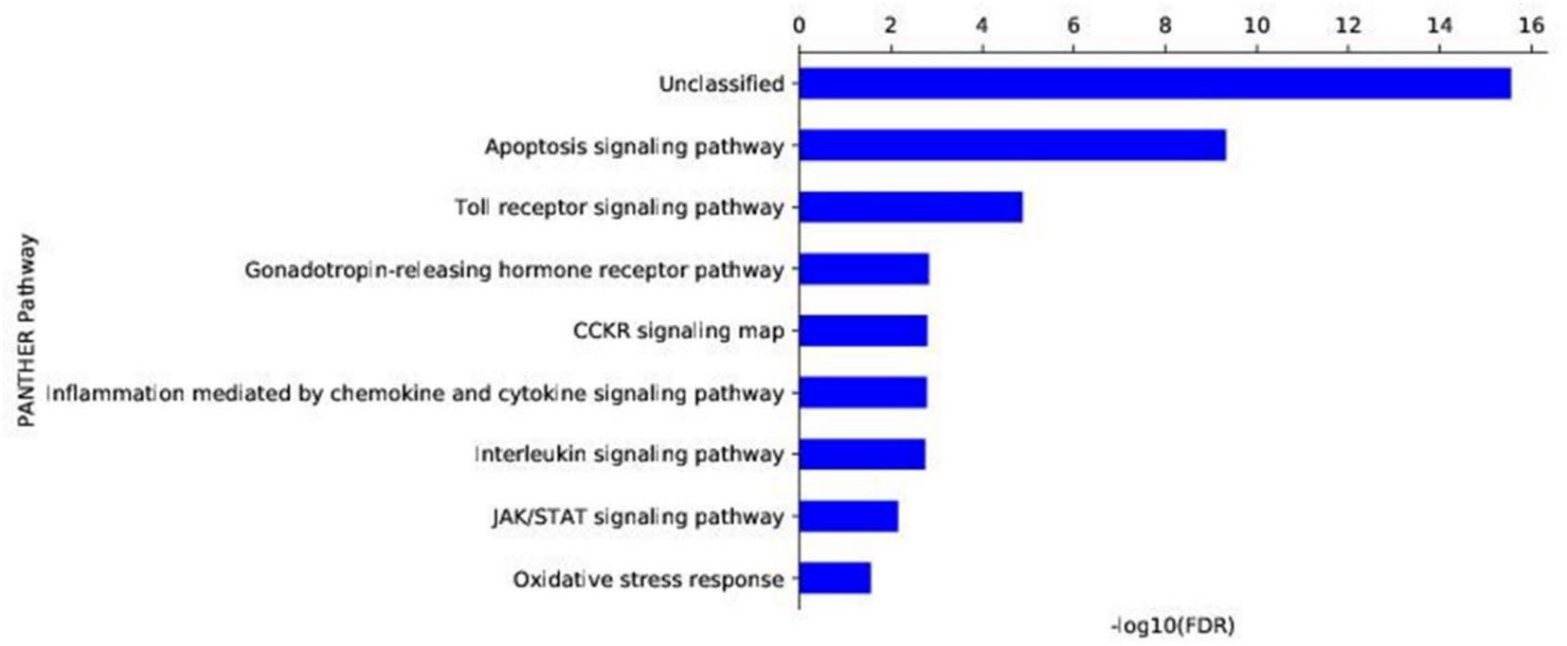
Pathways affected by LPS-stimulation of THP1-MΦ. RNAseq data was analysis using Panther-based data analysis to identify different signaling pathways that are affected by LPS-stimulation of macrophages.

In addition to gene expression analysis, we also analyzed the different pathways that are potentially affected upon LPS stimulation of THP1-MΦ using Panther-based database analysis. These analyses demonstrated that highly affected pathways include Toll like receptor (TLR) signaling, gonadotropin-releasing hormone receptor pathways, inflammation mediated chemokine and cytokines signaling pathways, interleukin signaling pathways and others (Fig. 3, Table 2 lists some of the genes affected in respective pathways. Notably, TLRs are crucial players in inflammation and immune response^65–68^. TLRs are class of pattern recognition receptors (PRRs) which recognize a variety of pathogen-associated molecular patterns (PAMPs) and danger associated molecular patters (DAMPs) derived from various pathogens and trigger downstream signaling, induction of cytokines and pro-inflammatory genes resulting in inflammation, macrophage activation and immune response. Similar to TLR signaling, gonadotropin-releasing hormone receptor, chemokine and cytokines signaling, and interleukin signaling pathways are also closely integrated with inflammation response and immune signaling^69,70^. These analyses further demonstrated that LPS-treatment induced the inflammatory response in THP1-derived macrophages.

**Table 2:** LPS-induced inflammation associated signaling pathways and genes expression.

Gene	Log2 fold change
Apoptosis signaling	
BIRC3	9.23
TNF	8.11
NFKB2	5.35
NFKBIA	5.26
BCL2A1	4.94
NFKB1	4.49
RELB	4.00
MAP2K3	3.88
CFLAR	3.58
TNFSF10	3.57
Toll receptor signaling	
NR4A1	7.99
SRC	6.57
BMP6	544
MAP3K8	5.27
SMAD1	4.92
PTGIR	3.98
ATF3	3.89
MAP2L3	3.88
JUNB	3.42
PTGER4	3.07
Gonadotropin-releasing hormone	
TNFAIP3	6.04
PTGS2	5.95
NFKB2	5.35
MAP3K8	5.27
NFKBIA	5.26
IFNB1	4.75
NFKB1	4.49
IRF7	3.90
MAP2K3	3.88
MYD88	2.65
Gonadotropin-releasing hormone	
NR4A1	7.99
SRC	6.57
BMP6	5.44
MAP3K8	5.27
SMAD1	4.92
PTGIR	3.98
ATF3	3.89
MAP2K3	3.88
JUNB	3.42
PTGER4	3.07
CCKR signaling map	
BIRC3	9.29
NR4A1	7.99
CXCL8	6.68
SRC	6.57
CXCL1	6.44
PTGS2	5.95
IER3	5.28
NFKBIA	5.26
CETP	4.59
SNAI1	3.10
Inflammation mediatedby chemikines	
CCL3L3	11.13
CCL8	9.82
CCL3	9.20
IL6	3.71
IL1B	7.52
BCL3,AC092066	6.96
CXCL10	6.80
CCL20	6.71
CXCL8	6.68
GNG4	6.27
Interleukin signaling	
IL6	8.71
CXCL8	6.68
IL23A	6.51
STAT4	6.26
ILIA	4.94
IL15RA	4.39
IL15	3.71
IL10RA	3.52
STAT5A	3.50
STAT2	3.02
Oxidative stress response	
DUSP8	5.21
DUSP2	5.00
MAP2K3	3.88
DUSP1	3.52
DUSP16	3.21
AC067945,STAT1	2.58
MYC	2.05
DUSP4	1.99
TXNL1	1.40
JUN	1.25
JAK STAT signaling	
STAT4	6.26
SOCS1	4.88
STAT5A	3.50
STAT2	3.02
AC067945,STAT1	2.53
JAK1	1.10
STAT3	1.10

Interestingly, our analysis demonstrated that along with well-known protein coding genes, cytokines and chemokine, LPS-stimulation of THP1-MΦ, also significantly affected (induction and down regulation) the expression of many noncoding RNAs. The highly upregulated long-noncoding RNA transcripts include ADORA2A-AS1 (ADORA2A antisense transcript 1), AC007362.3, AP001610.5, RP11-519G16.3, RP1-68D18.4, and many others (Fig. 2). We termed this novel of class of noncoding transcripts as Long-noncoding inflammation associated RNAs, LinfRNAs. We labelled these human LinfRNAs (hLinfRNA) as hLinfRNA1 (ADORA2A-AS1), hLinfRNA2 (AC007362.3), hLinfRNA3 (AP001610.5), hLinfRNA4 (RP11-519G16.3), hLinfRNA5 (RP1-68D18.4) and others as their order of upregulation (Log2 fold change) in the RNAseq analysis. Significant induction of hLinfRNAs along with protein coding genes and cytokines suggest their potential association with inflammation, macrophage activation, and immune response. However, it’s important to note that though these hLinfRNAs are identified as a transcript induced by LPS and they are potentially involved with inflammation signaling, their detailed structures and functions mostly remain elusive.

### hLinfRNAs expressions are induced by LPS

**Figure 4:**
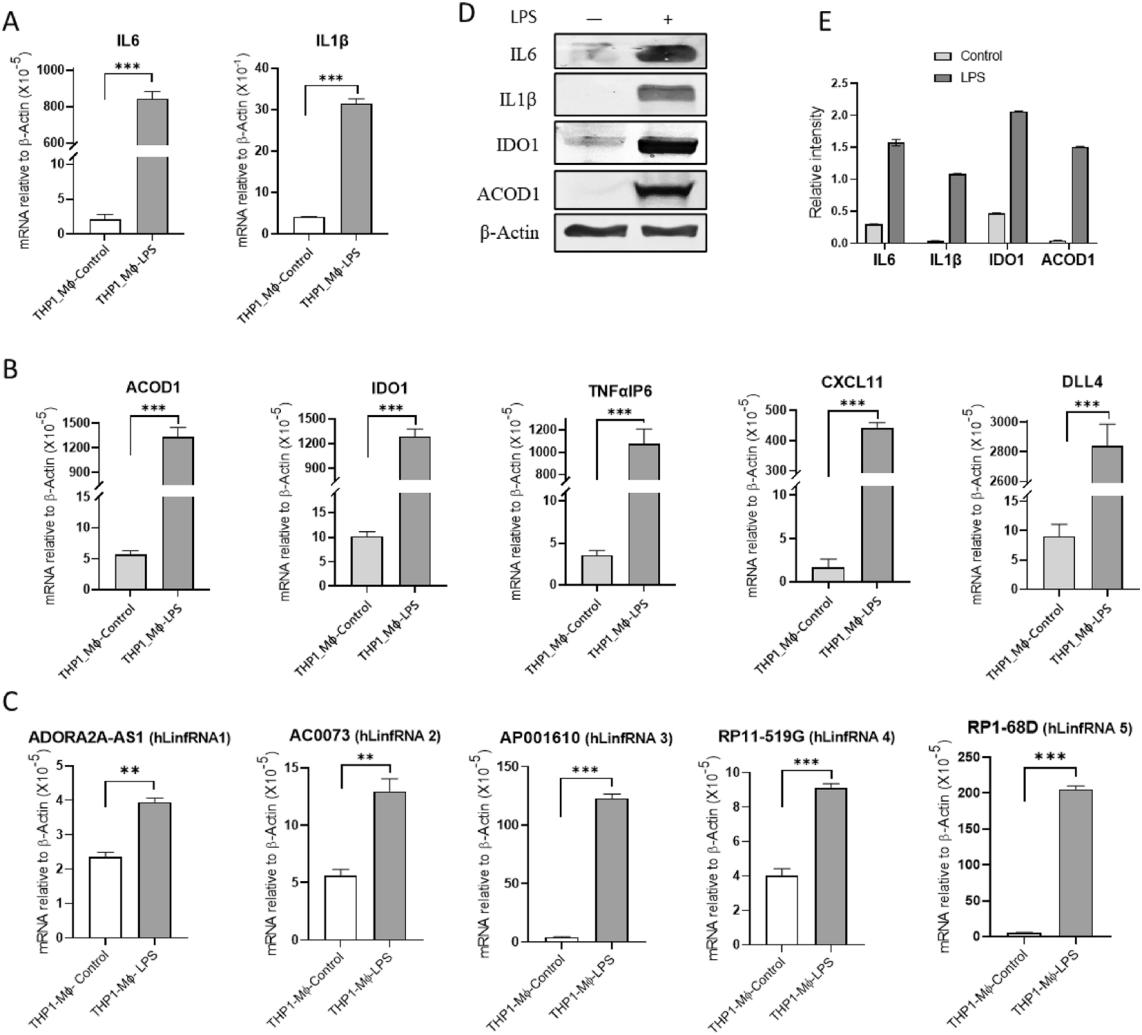
LPS induces inflammation in THP1-macrophages (THP1-Mɸ). THP1-Mɸ cells were treated with LPS (1 μg/mL, 4 h), total RNA and proteins were isolated. RNA was reverse transcribed to cDNA and analyzed by RT-qPCR for expression of proinflammatory cytokines like IL-6 and IL-1β (**A**), as well as top upregulated protein coding genes (found in RNA-seq analysis) including ACOD1 and IDO1 at transcript level (**B**); and hLinfRNAs (1–5) (**C**). (**D**) Western blot analysis of protein coding genes. Proteins from the control and LPS-treated THP1-MΦ were analyzed by Western blot using antibodies against IL6, IL-1β, ACOD1, IDO1, and β-Actin (control). Bands were quantified and plotted in Fig. 4E. The specific region selected for each western blot is shown by red–rectangle in original respective western blot in the supplementary figure S1. Each experiment was repeated at least thrice with three parallel replicates. β-Actin was used as loading control. Data represent mean ± SEM (n = 3); *p < 0.05, **p < 0.001, ***p < 0.0001.

To further confirm the LPS-induced expression of coding and noncoding transcripts, we performed RT-qPCR analysis for the above highly upregulated coding and noncoding transcripts in the control and LPS-treated THP1-MΦ cells. As seen in Fig. 4, LPS-treatment induced the expression (mRNA levels) of well-known cytokines such as IL6 (425 fold) and IL1β (20 fold), coding genes ACOD1 (700 fold) and IDO1 (240 fold), TNFαIP6 (TNFα inducible protein 6; 300 fold), CXCLL11 (chemokine; 270 fold), DLL4 (300 fold) (Figs. 4A-B). LPS-treatment also induced the noncoding transcripts such as LinfRNA1 (ADORA2A-AS1; 1.7 fold), hLinfRNA2 (AC007362.3; threefold), hLinfRNA3 (AP001610.5; 30 fold), hLinfRNA4 (RP11-519G16.3; 2.3 fold), and hLinfRNA5 (RP1-68D18.4; 40 fold) (Fig. 4C). In addition to the RNA level, we also analyzed the protein level expression of well-known pro-inflammatory genes IL6 and IL1β, and top protein coding genes such as ACOD1 and IDO1 using western blot (Fig. 4D, quantification in Fig. 4E, and supplementary Figure S1). Beta-actin was used as a loading control. Interestingly, the LPS treatment indeed induced the expression of IL6, IL1β, ACOD1, and IDO1 in protein levels. The LPS induced expression of pro-inflammatory cytokines (IL6, and IL1β), protein coding genes (e.g. ACOD1, IDO1 and others), and noncoding transcripts such as hLinfRNAs (#1–5), and others, suggest that hLinfRNAs are potentially associated with inflammation and immune response in macrophages. Thus, along with many protein coding genes, our RNAseq analyses led to the discovery of a series of human specific LinfRNAs (hLinfRNAs) that are potential regulators of inflammation and the immune response in humans.

### LPS-induced expression of hLinfRNAs at varying doses and varying time of LPS treatments

**Figure 5:**
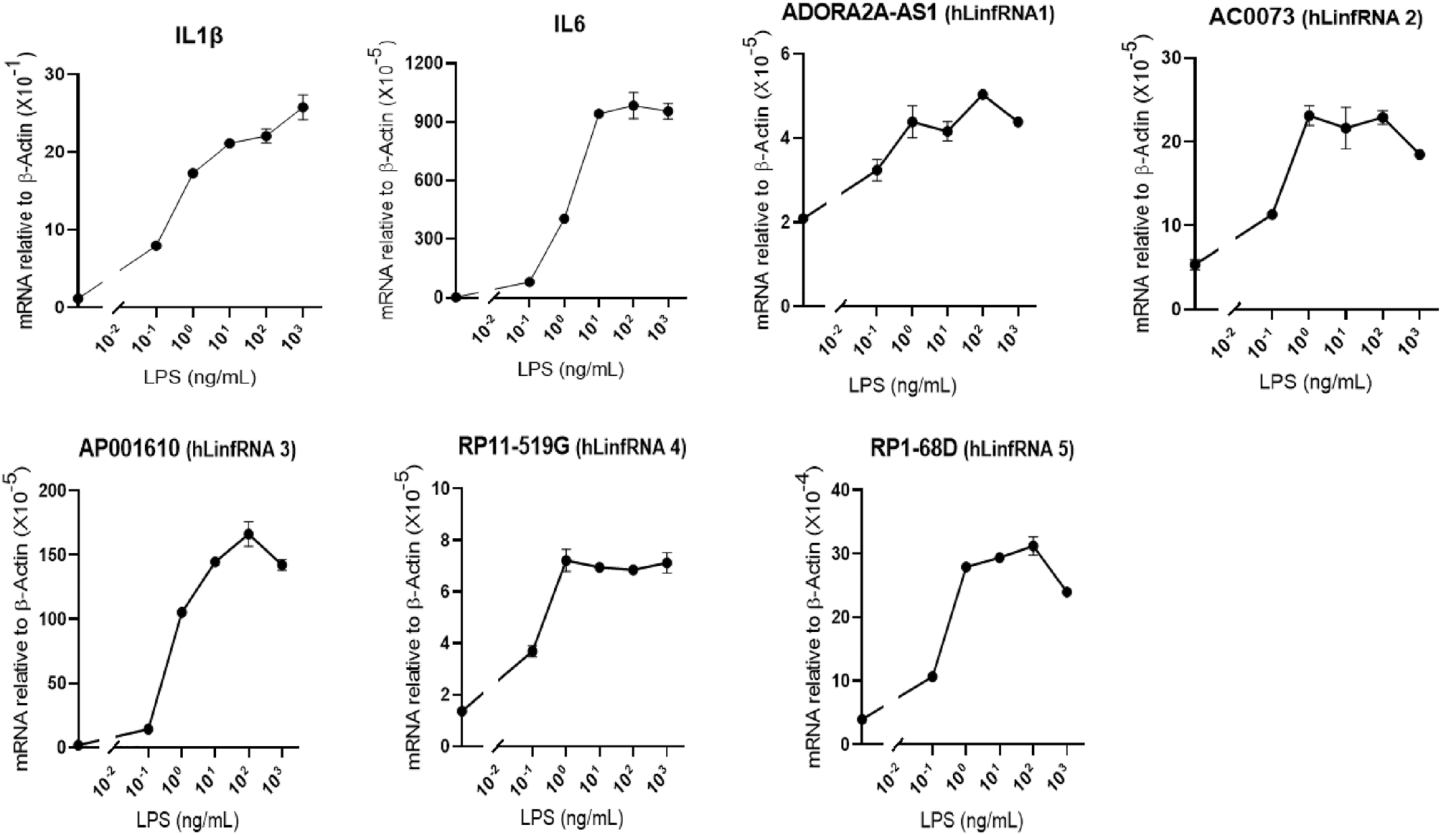
hLinfRNAs are expressed in a dose-dependent manner in THP1-macrophages (THP1-Mɸ) under LPS induced inflammation. THP1-MΦ cells were treated with varying concentration of LPS (0.1- 1000 ng/mL, 4 h), total RNA was isolated and analyzed by RT-qPCR for expression of proinflammatory cytokines (IL6, IL-1β) and top 5 hLinfRNAs. β-Actin was used as loading control. Data represents mean ± SEM (n = 3).

To further understand the potential association of LinfRNAs with inflammation, we examined their LPS-induced expression at varying concentration of LPS and varying time of LPS-treatments and compared that with well-known inflammatory markers such as IL6 and IL1β. Briefly, THP1-MΦ cells were treated with varying concentrations of LPS (0.1–1000 ng/mL, for 4 h) or for a varying time period ( up to 8 h) with 1000 ng/mL of LPS treatment. RNA was analyzed by RT-qPCR for the expression of LinfRNAs along with IL6 and IL1β. Dose-dependent analysis showed that the expression of IL6 and IL1β were increased with the increase in concentration of LPS and reaching a plateau at around 10–100 ng/mL and similar effects were observed for hLinfRNAs with some variations for different hLinfRNAs (Fig. 5). In particular, hLinfRNA1 (2.5 fold), hLinfRNA2 (fourfold), hLinfRNA4 (fourfold) and hLinfRNA5 (sevenfold) were induced by 2.5. 4, 4 and sevenfold respectively in response to LPS-treatment and reached a plateau at 1 ng/mL LPS (Fig. 5). Although, the hLinfRNA3 shows more dose dependent response and reaches the plateau around at 10 ng/mL like IL6. To be consistent for different genes, we used 1000 ng/mL LPS treatment for further studies.

**Figure 6:**
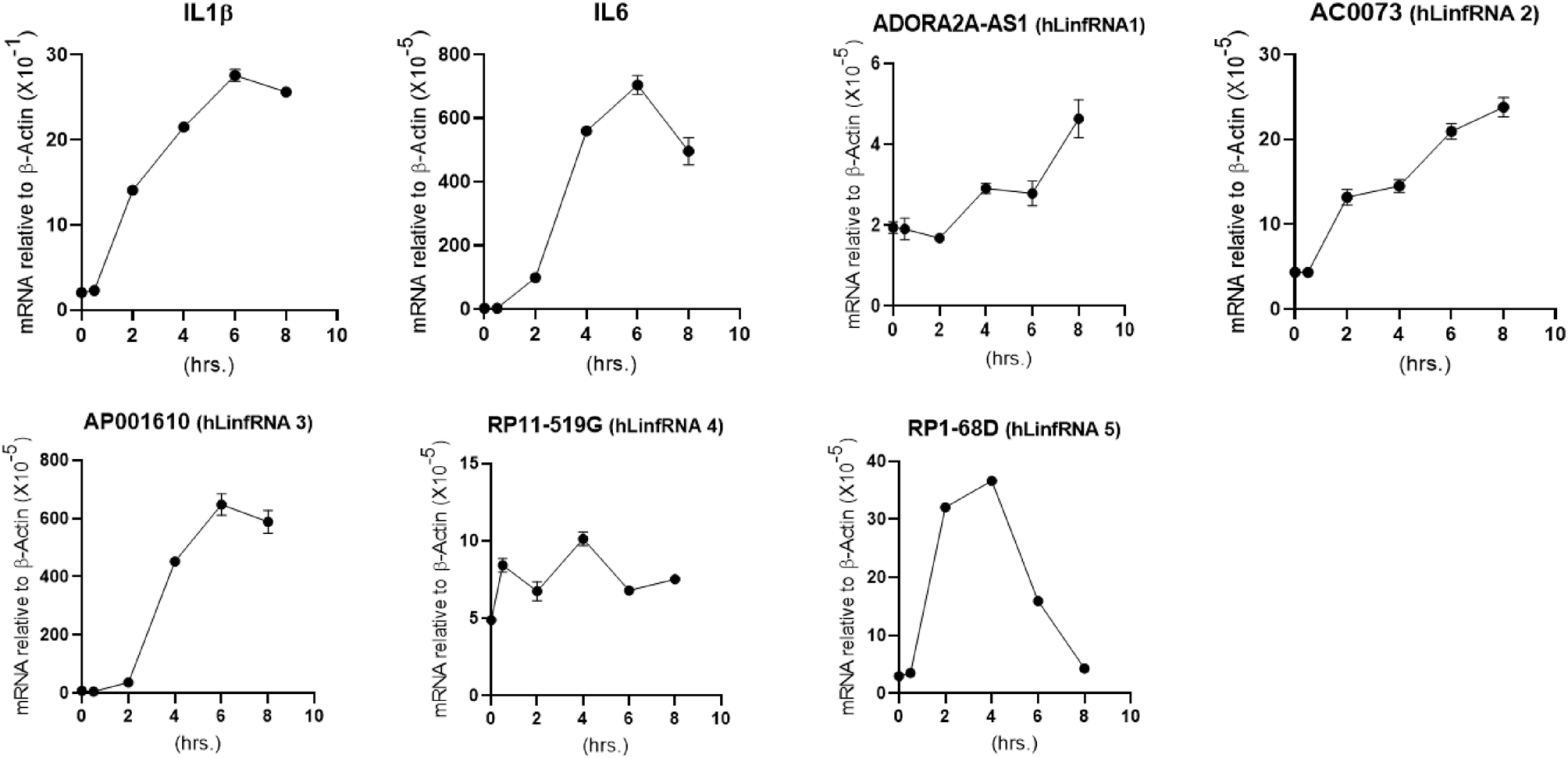
Temporal expression of hLinfRNAs under LPS-stimulation of THP1-Mɸ. THP1-Mɸ cells were treated with LPS (1 μg/mL) for varying time periods. RNA was analyzed by RT-qPCR for expression of proinflammatory cytokines (IL6, IL-1β) and top 5 hLinfRNAs. Each experiment was repeated at least thrice with three parallel replicates. β-Actin was used as loading control. Data represents mean ± SEM (n = 3).

Temporal studies demonstrated that IL6 and IL1β were increased with time reaching a maximum around 6 h and then decreased (Fig. 6). Interestingly, similar to IL6 and IL1β, LinfRNA3 induction was increased with time reaching a maximum around 6 h and then decreased suggesting similar type of expression behavior of LinfRNA in comparison to the IL6 and IL-1β (Fig. 6). hLinfRNA4 expression followed is increased with time and potentially plateaued at round 6–8 h of LPS treatments. hLinfRNA5 is relatively early responsive, while hLinfRNA1 may be late responsive compared to IL6 (Fig. 6). Thus, even though hLinfRNAs expression are induced by LPS-stimulation of THP1-MΦ, each RNA may have distinct modes of regulation during inflammation and macrophage activation and this behavior is similar to many inflammation associated protein coding genes, cytokines and chemokines.

### *LinfRNAs expressions are regulated *via* NF-κB*

**Figure 7:**
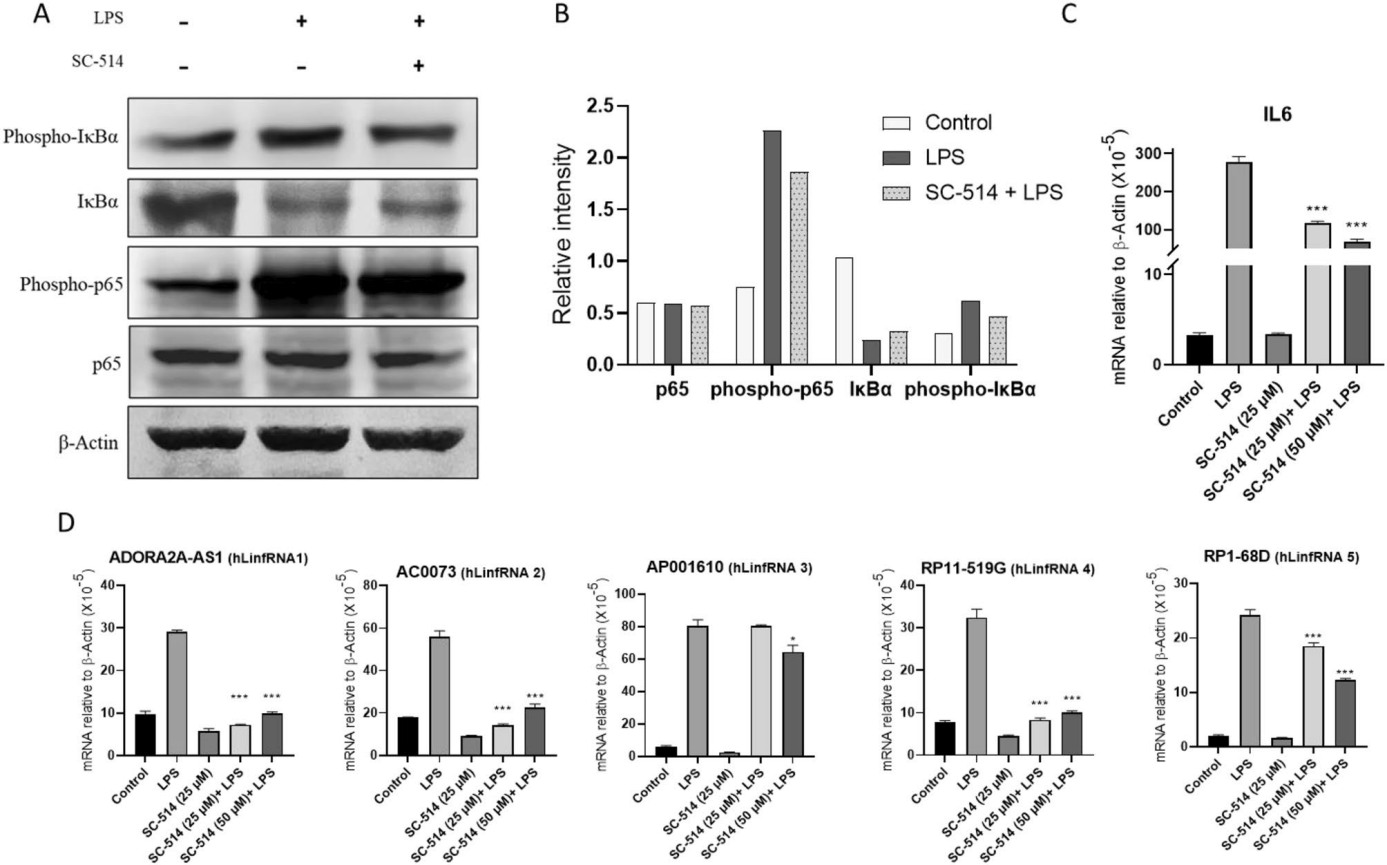
hLinfRNAs are regulated by NF-κB signaling pathway in THP1-macrophages. THP1-MΦ cells were treated with IKKβί (SC-514, 25 μM, 1 h) followed by LPS (1 μg/mL). RNA and proteins were isolated from the control and LPS (with and without SC514) -treated cells and analyzed by RT-qPCR and Western blotting respectively. (**A-B**) Western blot analysis for the IκBα, phospho-IκBα, p65 and phospho-p65 (β-actin was used as a loading control). Quantifications are shown in panel 7B. The specific region selected for each western blot are shown by red–rectangle in the original respective western blots, supplementary figure S2. C-D) RT-qPCR analysis for the expression of pro-inflammatory cytokine (IL6, panel **C**) and hLinfRNAs (1–5, panel **D**). Data represents mean ± SEM (n = 3). *p < 0.05, **p < 0.001, ***p < 0.0001.

Inflammation signaling is complex and may follow diverse pathways. Upon infections, pathogen associated PAMPs/DAMPs activate variety of PRRs (e.g. TLRs)^65–68^ that triggers signaling cascades and activates transcription factors such as NF-κB, AP1, IRFs, STATs, and induce transcription of pro-inflammatory genes (cytokines, chemokines, IFNs etc.) leading to inflammation response. LPS is well known to trigger TLR (TLR4 in particular) signaling and follow NF-κB activation. Here, initially, aimed to investigate if LinfRNA expressions are regulated by NF-κB activation. Briefly, THP1-MΦ cells were treated with IKKβ (IκB-kinase) inhibitors SC514 (25 and 50 µM, 1 h) followed by stimulation with LPS (1 μg/mL, 4 h), as described by us previously^32,33^. Notably, IKKβ is a kinase that phosphorylates IκBα (which inhibits NF-κB)^71–74^. Thus, the inhibition of IKKβ results in deactivation of NF-κB^73,74^. Thus, treatment with SC514 will result in inhibition of NF-κB activation and suppress inflammatory response. RNA and proteins from the control and SC514 treated (+ /− LPS-stimulation) THP1-MΦ cells were analyzed by RT-qPCR and Western blot for the expression of inflammatory genes and hLinfRNAs. Western blot analysis showed that LPS-treatment induced phospho-p65 (NF-κB subunit) as well as the phospho- IκBα level in comparison to the untreated control (Fig. 7A, quantification in 7B, supplementary figure S2), this LPS-induced increased phospho-p65 (NF-κB subunit) and the phospho- IκBα level was down-regulated upon treatment with SC514, suggesting LPS-induced activation of NF-κB and its deactivation by SC514. Notably, the total p65 level was mostly unchanged upon LPS or SC514 treatment, however, the IκBα level was reduced by LPS suggesting its degradation and this was inhibited by SC514 treatment (Fig. 7A, quantification in 7B, supplementary figure S2). The RNA from the control, SC-514 and LPS-treated cells were also analyzed by RT-qPCR for the expression of pro-inflammatory cytokine IL6. This analysis demonstrated that IL6 expression is induced by LPS-treatment, and this was significantly downregulated upon treatment with SC514, further demonstrating the SC514-mediated inhibition inflammatory response via inhibition of NF-κB activation (Fig. 7C). Interestingly, RT-qPCR analysis also demonstrated that LPS-induced expression of most hLinfRNAs (such as hLinfRNA1/2/4/5) were effectively suppressed by SC514, while expression of hLinfRNA3 was not affected (Fig. 7D). For example, similar to IL6, the expression of hLinfRNA1 (ADORA2A-AS1) was elevated (threefold) by LPS and treatment with SC514 down-regulated the LPS-induced elevation of hLinfRNA1 to almost basal level (Fig. 7D). Similar impacts of SC514 were observed for hLinfRNA2 and hLinfRNA4. (Fig. 7D), where LPS-induced expression was down-regulated to the basal level in presence of SC514-treatments. The basal level of expression (in the absence of LPS-treatment) of most hLinfRNAs were also suppressed by SC514-treatment. The LPS-induced expression of hLinfRNA5 was also repressed by SC514 (25%), though to smaller extent in comparison to hLinfRNA1, 2 and 4 (Fig. 7D). In contrast to IL6 and hLinfRNAs1/2/4/5, the LPS-induced elevation of hLinfRNA3 is most remain unaffected upon treatment with SC514 (Fig. 7D), These observations suggest that LSP-induced hLncRNAs1/2/4/5 expression are potentially regulated by NF-κB activation in macrophages, while hLinfRNA3 is likely regulated by other mechanism.

### hLinfRNA1 knockdown downregulates pro-inflammatory cytokines expression under inflammation

**Figure 8:**
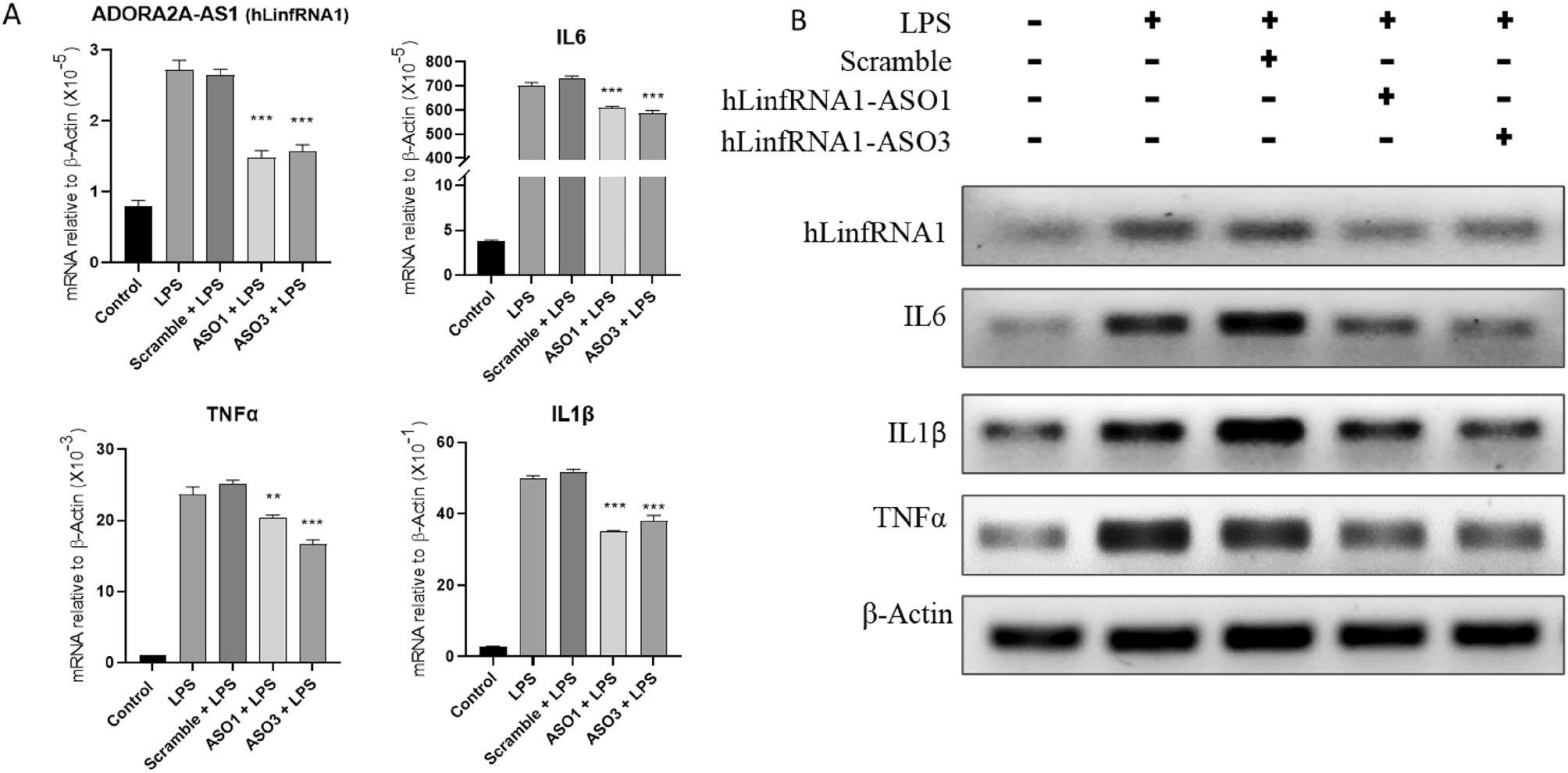
Knockdown of hLinfRNA1 down-regulates the LPS-induced inflammatory response in macrophage. THP1-MΦ cells were transfected with hLinfRNA specific antisense oligonucleotide (ASO1 and ASO3) and scramble antisense for 48 h, stimulated with LPS (1 μg/mL) and incubated for additional 4 h. RNA was analyzed by RT-qPCR for expression of hLinfRNA1 and proinflammatory cytokines IL6, TNFα and IL1β (Fig. 8A) and PCR amplified product was analyzed in 2% agarose gel electrophoresis (Fig. 8B). The specific region selected for each agarose gel is shown by red–rectangle in the supplementary figure S3. Each experiment was repeated at least thrice with three parallel replicates. β-Actin was used as loading control. Data represents mean ± SEM (n = 3); *p < 0.05, **p < 0.001, ***p < 0.0001.

To understand the potential function of LinfRNAs in inflammation, we knocked down one LinfRNA, hLinfRNA1 in macrophages and then analyzed its impacts on LPS-induced cytokine expression. To knockdown hLinfRNA1, we initially designed three antisense oligonucleotides (ASOs) for against hLinfRNA1 (Table 1) and tested for their knockdown efficacies, human beta-actin was used a loading control. hLinfRNA1-ASO1 and ASO3 showed effective knockdown efficacies of 50%) (Fig. 8A) and were used for additional experiments. Notably, hLinfRNA1 (NR_028484.3) is a 2831 nt long LncRNA with splice variants (NR_028483.2; 2052 nt), located in chromosome 22. Briefly, THP1-MΦ cells were transfected (48 h) with LinfRNA1-ASOs and scramble-ASO (control), independently, and then treated with LPS (additional 4 h). RNA was isolated and analyzed by RT-qPCR. Our analysis showed that hLinfRNA1 expression was induced by LPS and this was effectively knocked down (> 50%) upon transfection with hLinfRNA1-ASO1/ASO3 (Fig. 8A). Scramble-antisense has no significant impact on LPS-induced hLinfRNA1 expression. Interestingly, hLinfRNA1 knockdown down-regulated the LPS-induced expression of pro-inflammatory genes IL6, TNFα and IL1β significantly, suggesting critical roles of hLinfRNA1 in regulation of cytokines expression and inflammation in macrophage (Fig. 8A & 8B). Our observations demonstrated that hLinfRNA1 is not only regulated via NF-κB activation, but also is functional in regulation of NF-κB regulated cytokines expression during inflammation. Detailed roles of hLinfRNAs still remains elusive and our future goals.

## Discussion

According to the World Health Organization (WHO), infectious and inflammatory diseases remain a leading cause of death worldwide, with over 17 million new cases per year. Nearly 50,000 people die every day from infectious diseases^75^ because no treatments are available for many of these conditions. Sepsis, bowel disease, colitis, stroke, respiratory disease, obesity, diabetes, and cancer all have roots in chronic inflammation^76,77^. The prevalence of chronic inflammatory diseases is persistently increasing in the USA and around the world. Thus, understanding the mechanisms underlying inflammation and discovering novel regulators of inflammation are important for developing novel diagnostics and therapies.

NcRNAs are class of transcripts, which are encoded by the genome and transcribed, however, remains untranslated^78–83^. They are abundant in the cells and tissues and many of them are being detected in diseased cells, tissue, and circulating body fluids. NcRNAs are classified based on the sizes: small (< 50 nt), medium (50–200 nt), and long noncoding RNAs (lncRNAs, > 200 nt). Though, a large number of ncRNAs are being discovered, their structures and biochemical functions remains mostly unknown. NcRNAs, being nucleic acids, have the ability to interact with different proteins and other nucleic acids and thus, may contribute to alter protein and enzyme functions and may modulate cell signaling pathways, and ultimately may influence gene transcription, translation and gene expression processes.

Emerging evidence suggests that, along with protein-based factors, ncRNAs are also closely associated with inflammation and immune response. Our recent studies demonstrate that LncRNA HOTAIR plays key roles in cytokine regulation and inflammation in macrophages^32,33^. HOTAIR expression is upregulated in macrophages in response to LPS-induced inflammation. HOTAIR regulates NF-κB activation via regulation of IκBα degradation and hence regulates expression of cytokines and pro-inflammatory genes such as IL6, iNOS expression. Additionally, studies from our laboratory also demonstrated that HOTAIR regulates LPS-induced glucose transporter (Glut1) expression in macrophages and that in turn regulates the glucose uptake and metabolism. Notably, macrophages utilize glucose metabolism as a primary source of energy during inflammation. Thus, HOTAIR plays a critical role in regulation of inflammatory response and glucose metabolism in macrophages. Here, we aimed to discover novel lncRNAs that are critically linked to inflammation and immune signaling, in an unbiased manner. We performed an RNA-sequencing analysis in THP1-derived macrophages (THP1-MΦ) that were stimulated with LPS. Based on these analyses, we have discovered a series of novel human LncRNAs (termed as hLinfRNAs) that are potential regulators of immune response and inflammation. Similar to well-known protein coding genes and markers of inflammation, many hLinfRNAs are significantly up- and down-regulated upon LPS-induced inflammation in THP1-MΦ, suggesting their potential involved in macrophage activation, inflammation and immune signaling in human. Time course and concentration dependent LPS-stimulation induces these hLinfRNAs in macrophages. Thus, based on RNAseq analysis, we discovered a series of hLinfRNAs that are novel regulators of inflammation and immune signaling in humans. The functions of most of these LncRNAs remain elusive.

Inflammation and immune signaling are very complex processes and are associated with activation of macrophages and other immune cells^65^. The inflammation signaling may involve variety of pathways, receptors and factors. Notably, LPS in known to activate well known family of TLR receptors to induce inflammatory response. Among others, the transcription factor, NF-κB activation plays a central role in immune response and inflammation. NF-κB activation induces expression of NF-κB regulated cytokines and pro-inflammatory genes^65,84^. In the absence of any inflammation stimuli, NF-κB is complexes with I-κBα and remains inactive. However, upon inflammation signal, the I-κBα get phosphorylated followed by polyubiquitination and degradation. The degradation of I-κBα releases NF-κB (activated), which translocate to the nucleus, binds to the target gene promoters resulting in their induction in gene expression. As LPS-is well known to activate TLR signaling and NF-κB activation during inflammation, we investigated if newly discovered hLinfRNAs are potentially regulated via NF-κB signaling pathways. Towards, we applied well-known IKKβ inhibitor, SC514, which inhibits NF-κB. Importantly, application SC514 suppressed the LPS-induced expression of well-known cytokines and most hLinfRNAs (such as hLinfRNA1, 2, 4 & 5), suggesting their potential regulation via NF-κB signaling. Notably, there were few hLinfRNAs (such as hLinfRNA3) expressions were not affected upon NF-κB inhibition suggesting alternate mode of regulations.

The functions of most of these hLinfRNAs is unknown. To understand the potential roles of hLinfRNAs in inflammation, we knocked down the one of the hLinfRNAs, hLinfRNA1 (ADORA-2A-AS1), using anti-sense oligonucleotide (ASO) on in THP1-MΦ and analyzed LPS-dependent expression of cytokine expression. Interestingly, our results demonstrated that application of hLinfRNA1-ASO not only knocked the level of hLinfRNA1 expression, but also down-regulated the expression of well-known cytokines such as IL6, IL1β and TNFα. These observations demonstrate that hLinfRNAs are functional and are potential regulators of cytokines expression, inflammation, and immune response.

**Figure 9:**
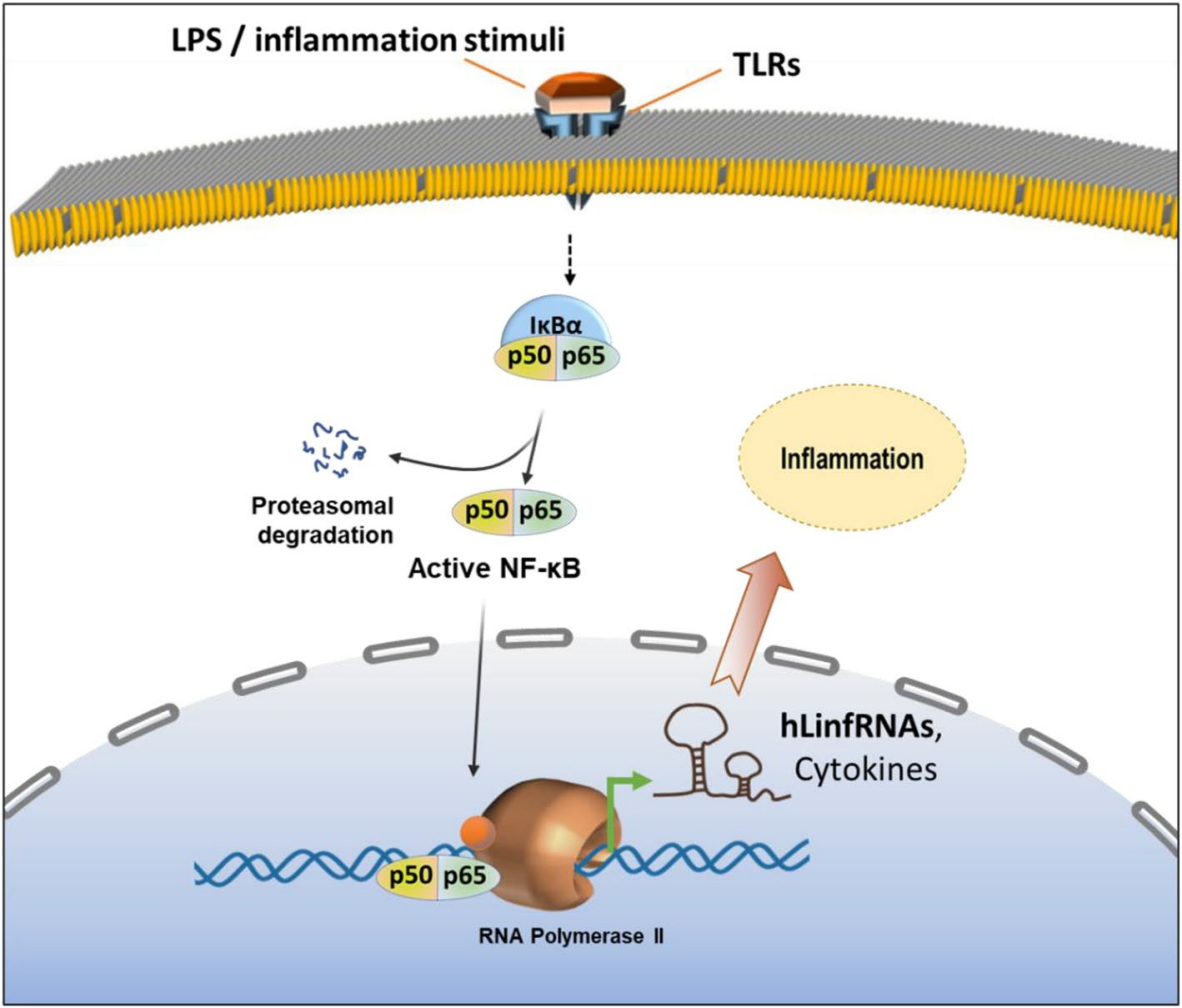
Model showing the induction of hLinfRNAs and their roles in inflammation. LPS induces TLRs activation and that triggers a cascade of downstream signaling including NF-κB activation and that induces expression of cytokines and pro-inflammatory genes. Our studies demonstrated that along with cytokines, LPS-induces the expression of a series of noncoding RNAs including LinfRNAs. LinfRNA expression are regulated via NF-κB signaling and potentially via other pathways. Knockdown of hLinfRNAs affect the expression of cytokines suggesting their functionality and potential roles in inflammation. Detailed structure functions of most hLinfRNAs remain elusive.

Independent studies from our laboratory and others demonstrate that lncRNAs plays critical roles in inflammation and immune signaling^32,85–91^ .LncRNA being long in sizes, have the ability interact with variety of proteins and transcription factors and modulate their activities and thus lncRNAs may influence enzymatic functions and cell signaling events. Our discovery of the series of hLinfRNAs suggests their potential functions in immune response and inflammation. Notably, the expression levels of different LinfRNAs vary and often it is much lower in comparison to well-known cytokines and pro-inflammatory genes expression. However, their time course and response behavior follow similar patterns to many cytokine expressions. Like cytokines and pro-inflammatory genes, most LPS-induced hLinfRNAs expression are regulated via NF-κB signaling pathways. Additionally, independent knockdown of hLinfRNAs altered the expression of well-known cytokines and suggested their functionality in inflammation, macrophage activation and immune signaling. Irrespective of their level of induction, each hLinfRNAs are unique and expected to have their own mode of regulation and functions. Overall, here we discovered a series of novel hLinfRNAs that are potentially regulators of inflammation and immune signaling. A model showing the induction of hLinfRNAs during macrophage activation and their potential roles in inflammation and macrophage activation is shown in Fig. 9. Like many other lncRNAs, the functions of hLinfRNAs remain unknown and will require a significant amount of time for their structural and functional characterization. The modes of action of different hLinfRNAs may vary. Similar to other nucleic acids, hLinfRNAs may interact with proteins and enzymes regulating their structures and functions and eventually contributing towards regulation of gene expression and regulation, cell signaling and metabolism, differentiation, growth and development. Many lncRNAs may act as a precursor to microRNAs or may also act as microRNA sponge and thus, may regulate mRNA stability, functions, and cell signaling events. Misregulation of hLinfRNA may contribute towards human diseases. Nevertheless, the discovery of the novel hLinfRNAs opens new avenues for screening their expressions in different types of inflammatory and immune diseases towards discovery LinfRNA-based biomarkers and therapeutic targets.

## Supplementary Information

Supplementary Information.

## Data Availability

All data generated or analyzed during this study are included in this published article [and its supplementary information files]. RNAseq data is available on https://www.ncbi.nlm.nih.gov/geo/query/acc.cgi?acc=GSE224561.
