# Supplementary material for: Novel long non-coding RNAs associated with inflammation and macrophage activation in human
Source: Sci Rep. 2023 Mar 10;13:4036. doi: 10.1038/s41598-023-30568-1 (PMC10006430; doi:10.1038/s41598-023-30568-1)
Supplement: Supplementary file 1 — Supplementary Information. [file 41598_2023_30568_MOESM1_ESM.pdf]

Supplementary information

Novel long non-coding RNAs associated with inflammation and macrophage activation in human

Avisankar Chini[1], Prarthana Guha[1], Venkat Malladi[3], Gibiao Zhao[2], and Subhrangsu S. Mandal[1]*

[1]Department of Chemistry and Biochemistry, The University of Texas at Arlington, Arlington, Texas 76019.

[2]North Texas Genome Center, The University of Texas at Arlington, Arlington, Texas 76019.

[3]Bioinformatics Core Facility, Lyda Hill Department of Bioinformatics, University of Texas Southwestern Medical Center, Dallas, Texas 75390.

Figure S1

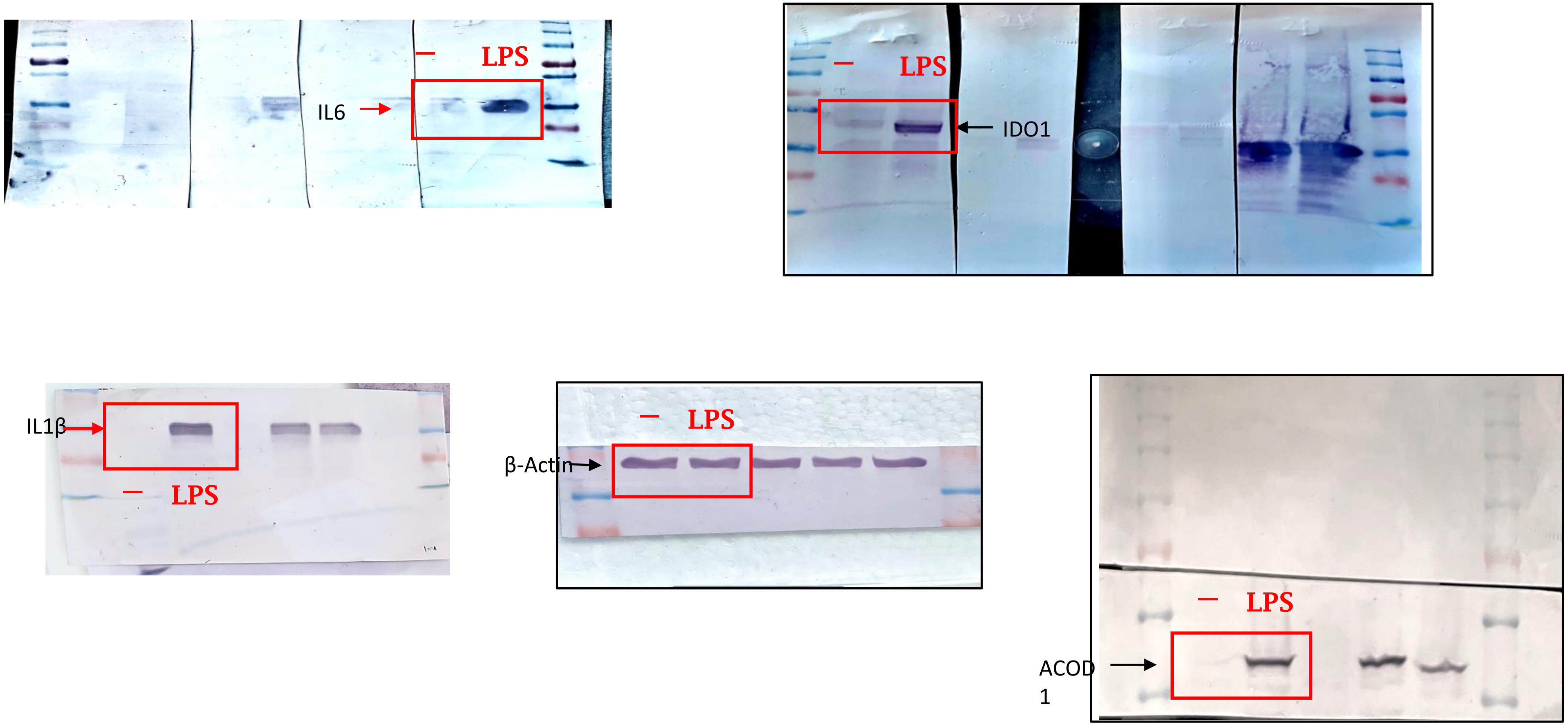

Figure S1. Western blot analysis of protein coding genes. Proteins from the control and LPS-treated (1 µg/mL, 6 h) THP1-MΦ were analyzed by Western blot using primary antibodies against IL6 (1:1000 dilution, GTX110527, GeneTex), and IDO1 (1:1000 dilution, 13268-1-AP, Proteintech), ACOD1 (1:1000 dilution, 775010S, Cell Signaling), IL1 β (1:1000 dilution, 16806-1-AP, Proteintech) and β-actin (1:1000 dilution, A2066, Sigma). For the secondary antibody treatment, membranes were incubated with AP-conjugated Goat anti-mouse (# ab97020, Abcam) or goat anti-rabbit secondary (# ab6722, Abcam) antibody and developed with BCIP-NBT (Alkaline phosphatase substrate, Promega) solution. (Images were taken with cell phone camera; quantified with ImageJ software normalizing with β-Actin as loading control).

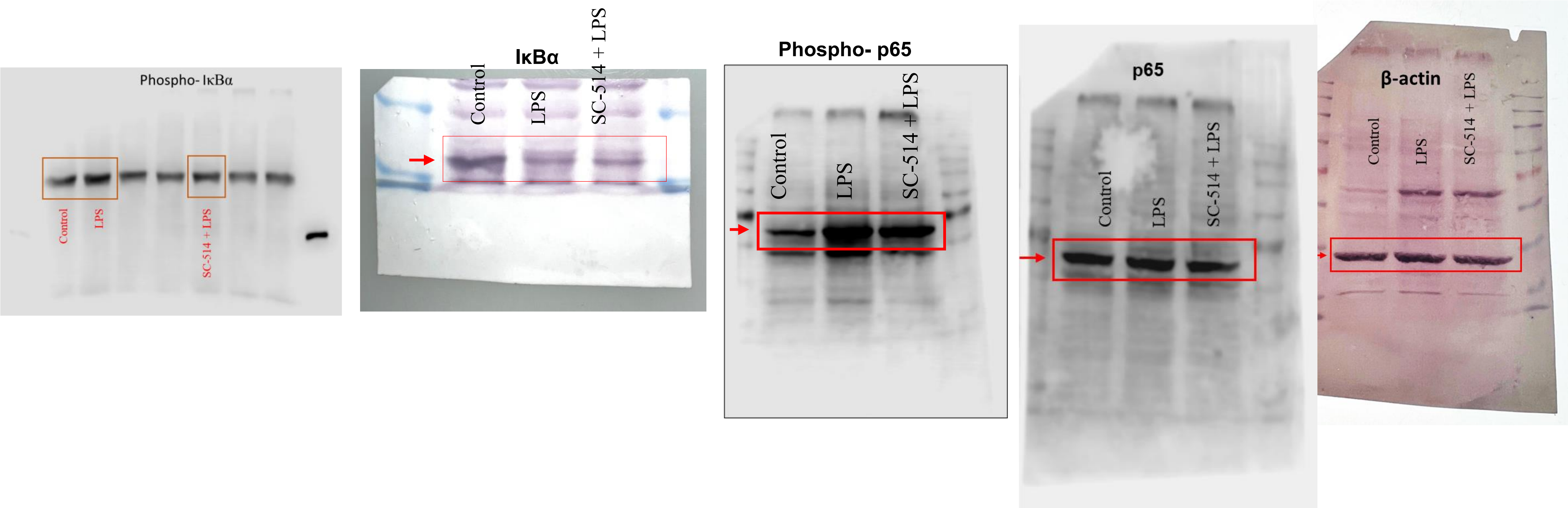

Figure S2. Western blot analysis of NF-kB inhibition assay. Proteins from control, LPS-treated (1 μg/mL, 1 h) and SC-514 (IkKβ$_{i,}$ 25 μM**)** + LPS (1 h + 1 h) treated THP1-MΦ were analyzed by Western blot using primary antibodies against Phospho-IκBα (1:1000 dilution, 2859S, Cell Signaling), IκBα (1:1000 dilution, 4814T, Cell Signaling), Phospho-p65 (NF-κB subunit, 1:1000 dilution, 3033S, Cell Signaling), p65 (1:1000 dilution, 10745-1-AP, Proteintech) and β-actin (1:1000 dilution, A2066, Sigma). For the secondary antibody treatment, membranes were incubated with AP-conjugated Goat anti-mouse (# ab97020, Abcam) or goat anti-rabbit secondary (# ab6722, Abcam) antibody and developed with BCIP-NBT (Alkaline phosphatase substrate, Promega) solution. For ECL Western blot, we used Horseradish peroxidase conjugated goat Anti-Mouse (# ab6789, Abcam) or goat Anti-Rabbit (# ab6721, Abcam) secondary antibodies and developed with developed using Pierce ECL western blotting substrate in LI-COR C-Digit blot scanner. Bands were quantified with ImageJ software and plotted.

Figure S3

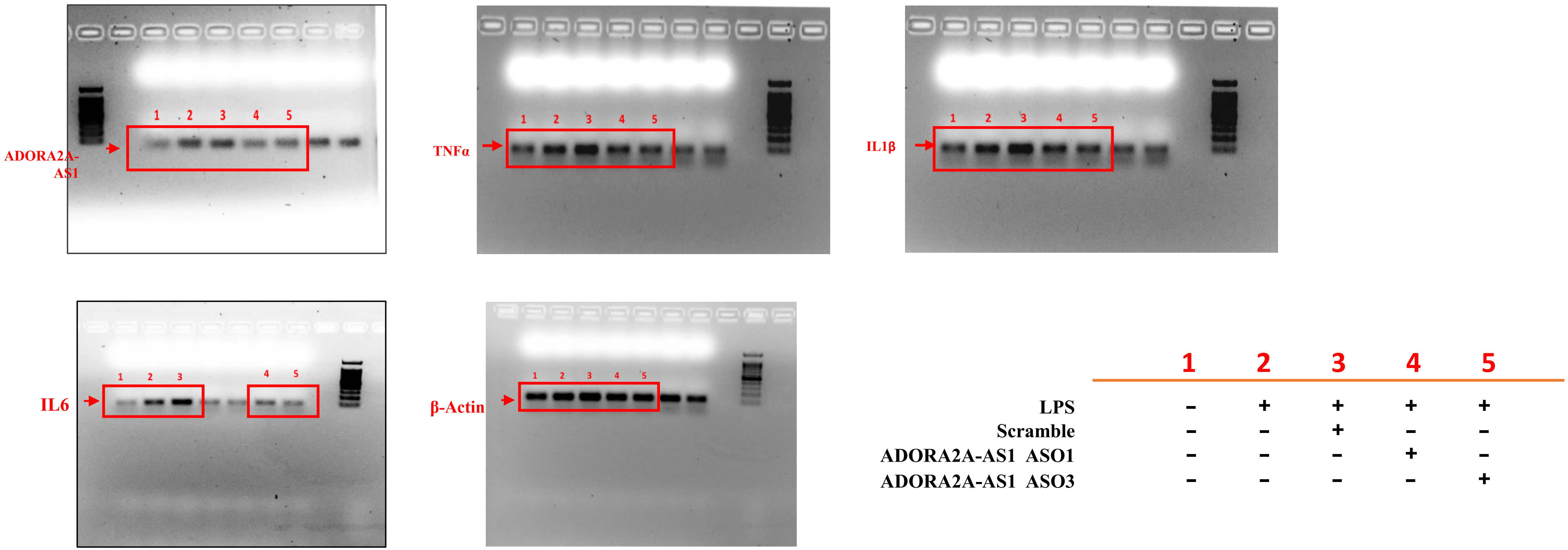

Figure S3. PCR analysis and agarose gel image of ADORA2A-AS1 (hLinfRNA1) knockdown. After RNA extraction (Trizol method), 1 µg of total RNA was reverse transcribed into cDNA and used as template for PCR (details in cDNA synthesis in methods section). Equal volume (6 µL) of cDNA was used as template and PCR amplified (semi-quantitative, total volume 20 µL) with gene specific qPCR primers for 35 cycles with following setup. 5 min initial denaturation (95 °C), in-cycle denaturation (95 °C for 30s), annealing (58 °C for 20s), polymerization/extension (72 °C for 30 s) and 2 min final extension (72 °C). 15 µL of PCR amplified product was mixed with 3 µL of 6X gel loading dye and run in 2% agarose gel at 140V for 40 min. Gel image was taken by BioRad GelDoc system with default setup.
